# Automated detection of hospital outbreaks: A systematic review of methods

**DOI:** 10.1371/journal.pone.0176438

**Published:** 2017-04-25

**Authors:** Brice Leclère, David L. Buckeridge, Pierre-Yves Boëlle, Pascal Astagneau, Didier Lepelletier

**Affiliations:** 1 Department of Medical Evaluation and Epidemiology, Nantes University Hospital, Nantes, France; 2 MiHAR laboratory, Nantes University, Nantes, France; 3 Department of Epidemiology, Biostatistics and Occupational Health, McGill University, Montreal, Canada; 4 UMR S 1136, Pierre Louis Institute of Epidemiology and Public Health, Pierre and Marie Curie University, Paris, France; 5 Department of Public Health, Pierre and Marie Curie University, Paris, France; 6 Centre de Coordination de la Lutte contre les Infections Nosocomiales Paris-Nord, Hôpital Broussais, Paris, France; 7 Department of Microbiology and Infection Control, Nantes University Hospital, Nantes, France; University Hospital Jena, GERMANY

## Abstract

**Objectives:**

Several automated algorithms for epidemiological surveillance in hospitals have been proposed. However, the usefulness of these methods to detect nosocomial outbreaks remains unclear. The goal of this review was to describe outbreak detection algorithms that have been tested within hospitals, consider how they were evaluated, and synthesize their results.

**Methods:**

We developed a search query using keywords associated with hospital outbreak detection and searched the MEDLINE database. To ensure the highest sensitivity, no limitations were initially imposed on publication languages and dates, although we subsequently excluded studies published before 2000. Every study that described a method to detect outbreaks within hospitals was included, without any exclusion based on study design. Additional studies were identified through citations in retrieved studies.

**Results:**

Twenty-nine studies were included. The detection algorithms were grouped into 5 categories: simple thresholds (n = 6), statistical process control (n = 12), scan statistics (n = 6), traditional statistical models (n = 6), and data mining methods (n = 4). The evaluation of the algorithms was often solely descriptive (n = 15), but more complex epidemiological criteria were also investigated (n = 10). The performance measures varied widely between studies: e.g., the sensitivity of an algorithm in a real world setting could vary between 17 and 100%.

**Conclusion:**

Even if outbreak detection algorithms are useful complementary tools for traditional surveillance, the heterogeneity in results among published studies does not support quantitative synthesis of their performance. A standardized framework should be followed when evaluating outbreak detection methods to allow comparison of algorithms across studies and synthesis of results.

## Introduction

Hospital information systems are goldmines for infection preventionists and epidemiologists. The large amount of data that they contain can help to detect adverse events, highlight risk factors, and evaluate the effectiveness of preventive actions [1]. These big data differ substantially from the ones that epidemiologists traditionally handle, but thanks to innovative methods borrowed from machine learning, data mining and natural language processing, they can be used to improve the quality and safety of healthcare [2]. Indeed, recent literature reviews have shown how these methods have been successfully applied to identify nosocomial infections [1,3], adverse drug events [4], and a wide range of other complications within hospitals [5].

Identifying nosocomial infections is useful to detect hospital outbreaks, which, given the potential morbidity, disorganization and cost that they can cause, represent a menace to patients, caregivers and healthcare systems [6,7]. However, case identification is only the first step in the surveillance process, and epidemiologists must then search for patterns that substantiate epidemic spread [8].

Fortunately, a wide range of automated outbreak detection methods is available and routinely used for community syndromic surveillance. Several infection control teams have also studied the usefulness of these methods at the scale of their own hospital, but the results were heterogeneous, precluding straightforward conclusions. The objective of our study was to clarify this issue by summarizing the existing literature on hospital outbreak detection algorithms, and especially by describing the evaluations approaches and the detection performances when applicable.

## Methods

### Study selection

In order to give the most accurate summary of the literature, we followed a systematic literature review protocol. The search query was built as a union of three sets of terms that related to the following key words: hospital, outbreak and detection (See S1 Appendix, protocol not accessible).

The MEDLINE bibliographic database was searched using the PubMed search engine in April 2016. To ensure the highest sensitivity, no limitations were imposed on publication dates and languages. The results of the query were screened based successively on the title, the abstract and the full text. Every reference that described a method used to detect outbreaks within hospitals was included, without any exclusion based on study design. The references that related to community outbreak detection or national/regional nosocomial infection surveillance were not included. The citations of every included document were also screened in search of relevant additional references, a method called *snowballing*. Complementarily, we performed *reverse snowballing* by identifying relevant documents that cited the included studies, using the Google Scholar search engine.

One author (BL) extracted data from the included studies using a standardized form. The variables of interest were the following: date of publication, country, study period, spatial and temporal scopes of the surveillance, events of interest, data sources, detection algorithms and evaluation methodology.

### Data analysis

To classify the studies according to their methodology, we used a framework developed by Watkins et al. for early outbreak detection evaluation [9]. According to this framework, four types of evaluation approaches can be identified: descriptive, derived, epidemiological, and simulation-based. The descriptive approach does not rely on detection performance measures, but rather on the description of detected events (frequency, duration, etc.). The “derived” approach uses the results of a statistical model as a reference standard to evaluate detection methods. The epidemiological approach uses more complex definitions based on multifactorial and flexible methods such as expert judgment. The last approach is the use of simulations, i.e. synthetic data. It allows for a complete control of outbreak features, but the validity of the estimations in the real world is not guaranteed. Besides classification, the methodologies were also analyzed to determine the risk of specific biases [10].

The performance measures (sensitivity, specificity, positive and negative predictive values) of the detection algorithms were also extracted, along with their 95% confidence intervals. If the confidence intervals were not available, we computed them based on the available data. The inter-algorithm heterogeneity was measured using the I^2^ statistics, which represents the percentage of variability that is due to heterogeneity between algorithms. As several studies used different algorithms, we also calculated an R^2^ statistic to estimate the proportion of the inter-algorithm heterogeneity that was due to differences between studies. These R^2^ were estimated using mixed-effect meta-regressions that included a study effect. All the statistical analyses were done using the R software version 3.2.0 and the metafor package.

The present article was prepared using the PRISMA checklist for items reporting in systematic reviews [11] (S2 Appendix).

## Results

**Fig 1:**
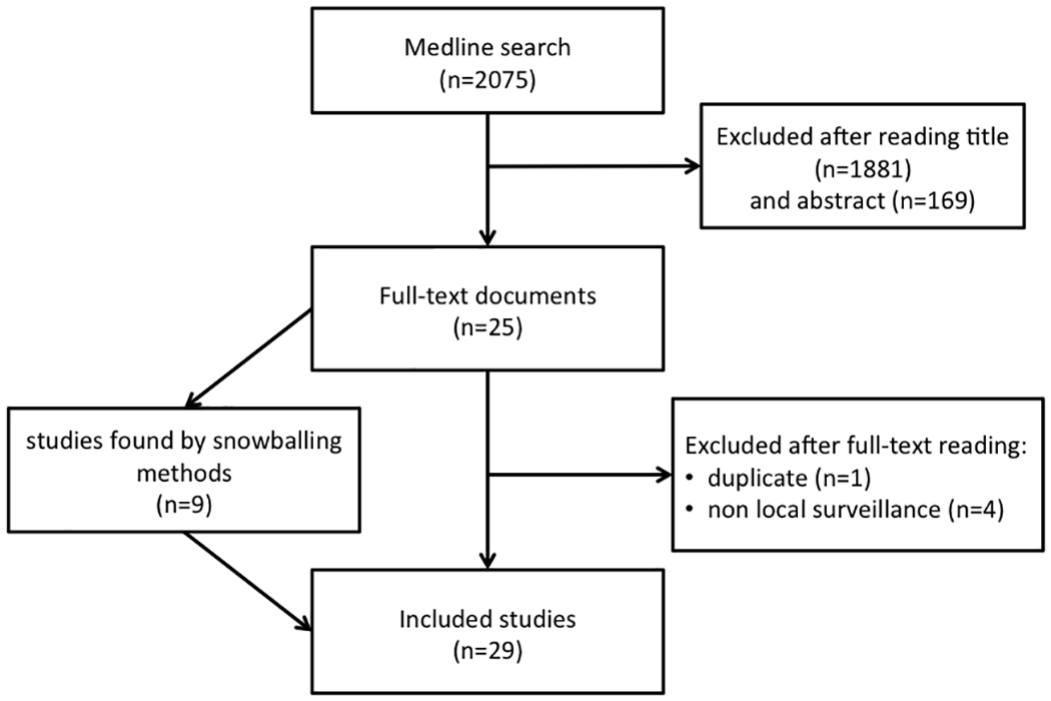
Study selection flow diagram.

Twenty-nine studies were included at the end of the selection process (Fig 1). They are described in details in Table 1. In the next sections, we will describe these studies with regards to the type of surveillance in which the algorithms were used and to the methods on which these algorithms relied. Finally, we will examine the observed performances for each evaluation approach.

**Table 1:** Description of the included studies.

Study	Country	spatial scope of surveillance	spatial stratification	time unit of detection frequency	monitored types of infection	monitored measures	data sources	type of detection algorithms	type of evaluation	length of evaluation in month
Childress and Childress (1981) [12]	USA	intensive care unit of a university hospital	not applicable	month	*Serratia marcescens* infections	number of isolates	bacteriological lab results	SPC (thresholds based on endemic rate)	descriptive	12
Dessau and Steenberg (1993) [13]	Denmark	university hospital	none	week	organism specific infections	number of isolates	microbiology lab results	statistical modeling (time series analysis)	descriptive	12
Mylotte (1996) [14]	USA	university long term care facility (120 beds)	none	month	location-specific nosocomial infections	number of cases	ICP surveillance	SPC (thresholds based on endemic rate)	descriptive	96
Brossette et al. (1998) [15]	USA	university hospital	none	month	*Pseudomonas aeruginosa* infections	proportion of cases	bacteriological lab results and patient demographics	data mining (association rules)	descriptive	12
Arantes et al. (2003) [16]	Brazil	pediatric intensive care unit of a university hospital	not applicable	month	nosocomial infections	incidence rate of cases	IC surveillance	SPC (u-chart)	descriptive	36
Sagel et al. (2004) [17]	Germany	tertiary-care hospital (900 beds)	none	week	MRSA infections	number of isolates	IC surveillance	SPC (c-chart)	descriptive	12
Pentland et al. (2006) [18]	USA	university hospital	none	day	MDR-GN infections	number of isolates	bacteriological lab results	scan statistics	descriptive	24
Lamma et al. (2006) [19,20]	Italy	university hospital	wards	week	organism specific infections	number of cases	bacteriological lab results	statistical modeling (time series analysis)	descriptive	
Menotti et al. (2010) [21]	France	university hospital	none	month	nosocomial invasive aspergillosis	number of cases	IC surveillance	SPC (CuSum, LC-CuSum)	descriptive	24
Gomes et al. (2011) [22]	Brazil	intensive care unit of a university hospital	none	week	nosocomial infections	number of cases	IC surveillance	SPC (CuSum, u-chart, EWMA)	descriptive	24
Freeman et al. (2013) [23]	England	hospitals participating in national surveillance	none	week	12 species-specific infections 7 MDRO infections	number of cases	national IC surveillance	statistical modeling (quasi-Poisson model) and SPC (CuSum and)	descriptive	36
Du et al. (2014) [24]	China	tertiary-care hospital (3500 beds)	wards	day	nosocomial infections	number of isolates, diarrhea cases or surgical site infections	automated nosocomial infection surveillance	simple thresholds (≥2 to 3 cases in 1 to 21 weeks)	descriptive	48
Faires et al. (2014)A [25]	Canada	community hospital (350 beds)	hospital, services and wards	day	*Clostridium difficile* infections	number of isolates	bacteriological lab results	scan statistics	descriptive	55
Faires et al. (2014)B [26]	Canada	community hospital (350 beds)	hospital, services and wards	day	MRSA infections	number of isolates	bacteriological lab results	scan statistics	descriptive	55
Lefebvre et al. (2015) [27]	France	2 university hospitals (1200 and 1800 beds)	Hospital and units	day	*Pseudomonas aeruginosa* infections	Number and incidence rate of isolates	bacteriological lab results	scan statistics	descriptive	112 and 78 (depending on the hospital)
Schifman and Palmer (1984) [28]	USA	university hospital (325 beds)	ward	month	organism and location specific infections	number of cases	ICP surveillance	simple thresholds (≥ 2 times the average culture rate)	epidemiological	6
Brossette et al. (2000) [29]	USA	university hospital	unit	month	organism, location and antibiotic resistance specific infections	proportion of isolates	bacteriological lab results	Data mining (association rules)	epidemiological	15
Brown et al. (2002) [30]	USA	tertiary-care pediatric facility (330 beds)	not applicable	isolate	MRSA and VRE infections	number of isolates	bacteriological lab results	SPC (CuSum, moving average)	epidemiological	69
Ma et al. (2003) [31]	USA	10 hospitals of a university medical center	unit	month	organism, location and antibiotic resistance specific infections	number of isolates	bacteriological lab results	Data mining (association rules)	epidemiological	3
Hacek et al. (2004) [32]	USA	university hospital (688 beds)	none	month	organism specific infections	number of isolates and incidence rate of isolates	bacteriological lab results	simple thresholds (100% increase in 2 month, ≥50% increase in 3 months) and SPC (Shewart chart)	epidemiological	96
Wright et al. (2004) [33]	USA	university hospital (656 beds)	hospital, service and ward	week	location, organism, type and resistance specific infections	number of isolates	bacteriological lab results and admission-discharge-transfer	SPC (user-definable control charts)	epidemiological	13
Huang et al. (2010) [34]	USA	university hospital (750 beds)	hospital, services and wards	day	31 organism specific infections	number of isolates	bacteriological lab results	scan statistics	epidemiological	60
Carnevale et al. (2011) [35]	USA	general and pediatric hospital (800 beds)	hospital and units	day	organism specific infections	number of isolates	bacteriological lab results	SPC (CuSum, EWMA), scan statistics, data mining (WSARE)	epidemiological	24
Nishiura (2012) [36]	Japan	not implemented	none	month	-	number of isolates	IC surveillance	statistical modeling (Poisson model)	epidemiological	-
Tseng et al. (2012) [37]	Taiwan	university hospital (2200 beds)	none	week	MDR organism infections	number of isolates	bacteriological lab results	SPC (control charts ± hierarchical clustering)	epidemiological	12
Mellmann et al. (2006) [38]	Germany	university hospital (1480 beds)	wards	week	MRSA infections	number of isolates	bacteriological lab results	simple thresholds (2 isolates in 2 weeks, ± molecular typing)	derived	60
Charvat et al. (2009) [39]	France	university hospital (878 beds)	none	day	bloodstream infections	number of cases	IC surveillance	simple thresholds (delay between cases)	derived	120
Kikuchi et al. (2007) [40]	Japan	prefectoral central hospital	wards	day	symptoms	number of cases	electronic medical records (symptoms)	statistical modeling (linear model)	simulation	15
Skipper. (2009) [41]	Danemark	university hospital	none	day	simulated	number of isolates	bacteriological lab results	statistical modeling (Poisson model)	simulation	

MRSA: methicillin resistant *Staphylococcus aureus*, VRE: vancomycin resistant *Enterococcus*, IC: infection control, MDR: multi-drug resistant, GN: Gram negative, SPC: statistical process control, EWMA: exponentially-weighted moving average, WSARE: ‘What’s Strange About Recent Events?’ algorithm, (LC-)CuSum: (Learning curve) cumulative sums.

### Surveillance scope

Across the studies, the algorithms were used to detect different types of events. Three studies aimed to detect every nosocomial outbreaks, without any additional precision regarding their size, duration or type [22,24,31]. In two other studies, the events corresponded to cases with a clinical definition, i.e. nosocomial invasive aspergillosis [21] and bloodstream infections [39]. The remaining studies focused on infections caused by specific organisms such as multidrug resistant (MDR) bacteria or organisms known to cause nosocomial infections. Additional data allowed some algorithms to be stratified by infection site (bloodstream, urinary tract, etc.), organism or resistance pattern [13–15,19,23,28,29,31,32,34,35,37].

In most of the included studies (n = 24), the surveillance was implemented at the level of an entire hospital, but larger and smaller scopes were also reported. One study was conducted in a health trust consisting of 10 hospitals [31], and another one examined hospital-level outbreak detection based on the national surveillance data in England [23]. Conversely, in three studies, outbreaks were monitored at the level of a single intensive care unit [12,16,22]. Additional data allowed in six studies to stratify outbreak detection at different spatial levels, from the whole hospital to services and units [25–27,33–35].

Nearly every algorithm relied on either bacteriological laboratory results (n = 17) or nosocomial infection counts estimated by active surveillance from the infection control team (n = 10). Two studies additionally extracted admission-discharge-transfer data to provide a denominator for computing incidence rates [27,33]. Kikuchi et al. tested a more syndromic approach: instead of positive cases, their algorithm relied on counts of new occurrences of symptoms found in the electronic medical records [40].

The periods covered by these data varied between studies from 3 to 120 months, with a median of 24 months (inter-quartile range: 12.25–58.75).

### Detection algorithms

**Fig 2:**
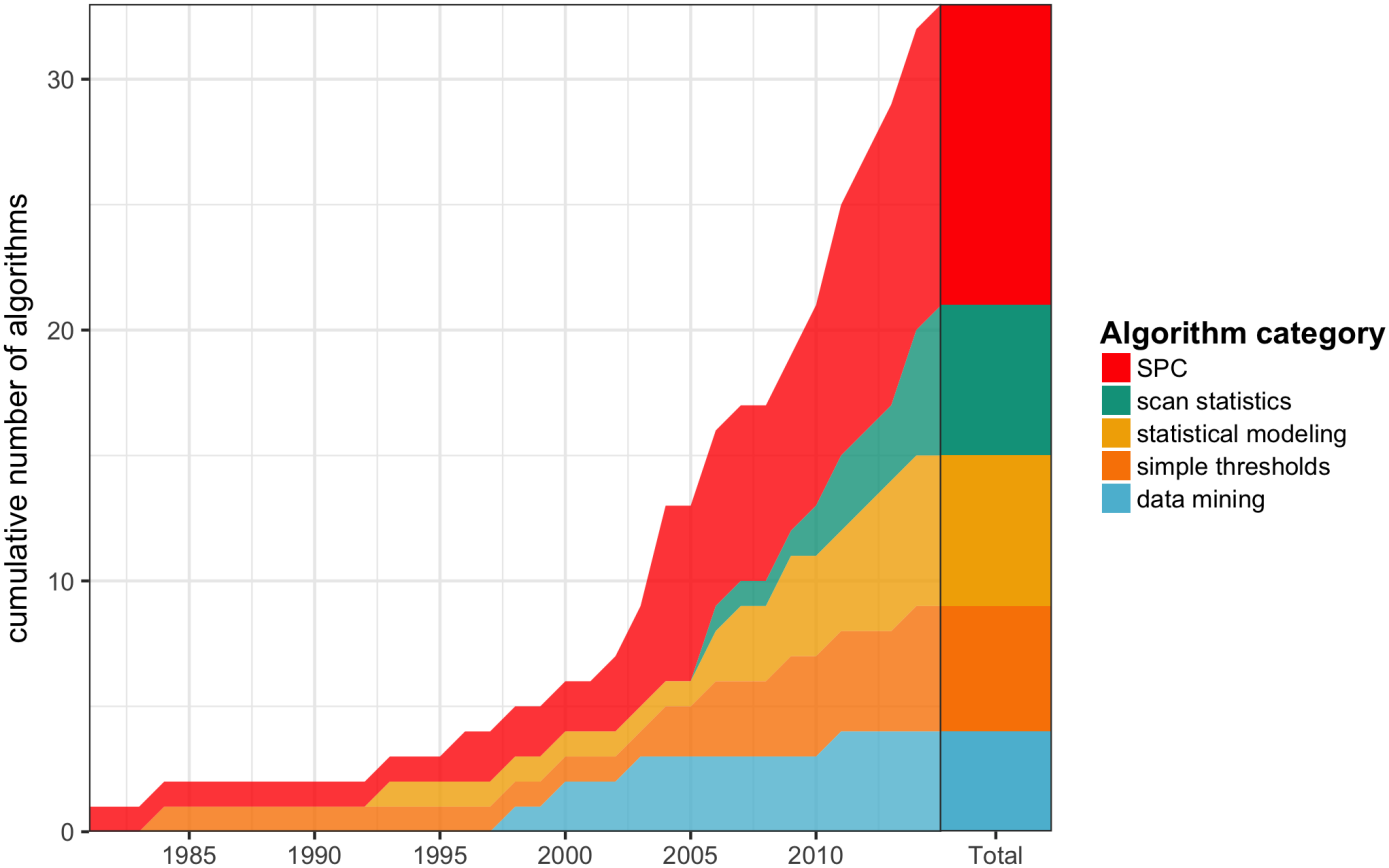
Cumulative count of detection algorithms found in the literature over time, by category. SPC: statistical process control.

Many different algorithms were implemented in the included studies, but they could all be classified into five categories: simple thresholds, statistical process control (SPC), statistical modeling, scan statistics and data mining methods. The trends of these categories over time are depicted in Fig 2.

With simple thresholds, an alert is triggered when the number of cases exceeded a threshold over which the number of infections in a given space and time is considered abnormal. These simple rules were used in six studies [24,28,32,34,38,39] and could either be an arbitrary threshold chosen by experts (e.g. three infections in two weeks in the same ward in the study by Huang et al. [27]) or a simple mathematical rule (e.g. a doubling of the ward’s average monthly culture rate in the study by Schifman and Palmer [28]).

Algorithms based on SPC were the most commonly used in the included studies (n = 12). For these algorithms, the alert threshold is not defined arbitrarily but based on statistical variations of cases frequency in the past. SPC offers the possibility to monitor different types of statistical parameter, such as incidence count or rate [14,16,17,22,28,32,37], cumulative sums (CuSums) [21,22,30,35] or moving averages [30,35,37].

Statistical models were used in six studies [13,19,23,36,40,41]. They mostly consisted of multivariate Poisson regressions that allowed taking into account predictable factors of fluctuation in the number of infection cases, such as seasonality.

Elaborating on these models, scan statistics represented another popular category of algorithms (n = 6) [18,25–27,34,35]. It even served as a reference standard in an additional study by Mellmann et al. [38]. Because they use adjustable surveillance windows, they are more flexible than traditional statistical modeling and can detect events at different space and time scales.

Data mining methods constituted the last category of algorithms, which was used in four studies. Three related studies used association rules to automatically detect interesting changes in infection occurrence and resistance patterns [15,29,31]. A third tested an algorithm called ‘What's Strange About Recent Events?’ (WSARE) [35], which relies on Bayesian networks and associations rules [42].

### Evaluation results

#### Descriptive approach

Fifteen of the included studies provided a descriptive evaluation of the algorithms’ results [12–19,21–27]. All of them showed that detection algorithms implemented in real hospital settings were able to generate relevant alerts, and some of them reported interesting additional observations. For example, Gomes et al. noted a complementarity between SPC algorithms: Shewart charts were better for detecting large deviations from the mean number of infections, while CuSums and exponentially-weighted moving averages were more suitable for detecting smaller deviations [22].

Freeman et al. noted that adding data about antibiotic resistance more than doubled the overall number of alerts generated by a Poisson-based model [23].

More recently, two studies by Faires et al. provided interesting insights about how a scan statistic-based algorithm compared to traditional infection control surveillance: it retrospectively identified most of the outbreaks already investigated by the infection control team but also flagged other potentially undetected epidemic events [25,26].

#### Epidemiological approach

The epidemiological approach was the second most frequently used evaluation design (n = 10). Its implementation, however, differed quite noticeably between studies. Some of them relied on the judgment of one [28,29,31] or several experts [30,33–35] to classify the alerts while others compared them to a list of previously identified outbreaks [36,37]. A last one used molecular typing, a common method for confirming clonal outbreaks, i.e. infections caused by the same strain [32].

**Fig 3:**
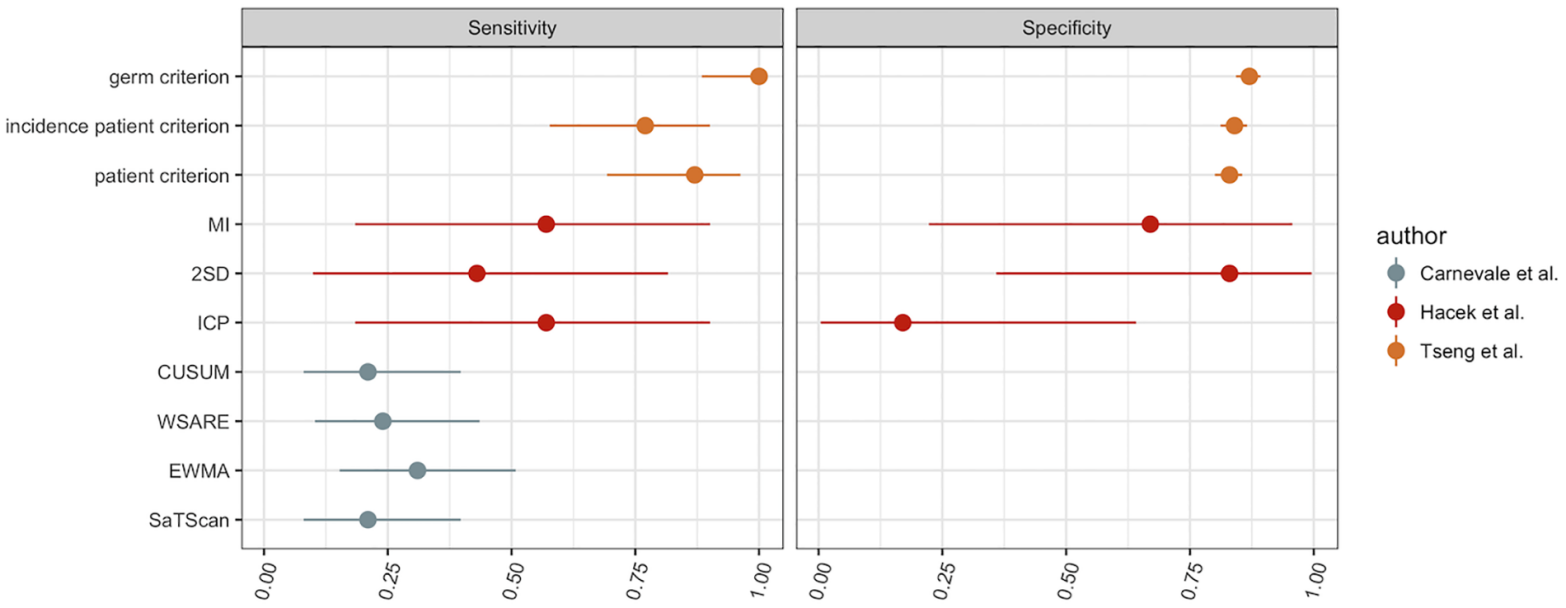
Sensitivity and specificity of the detection algorithms evaluated with the epidemiological approach (with 95% confidence intervals). Patient criterion: control chart based on the number of infected patients; incidence patient criterion: control chart based on the incidence of infected patients; germ criterion: control chart based on the number of positive results; MI: monthly increase; ICP: infection control surveillance; 2SD: control chart based on the number of positive results; WSARE: What’s Strange About Recent Events?; SaTScan: scan statistics; EWMA: Exponentially-Weighted Moving Average; CUSUM: Cumulative sum.

Experts’ evaluation of the alerts allowed the computation of positive predictive values (PPVs). As PPVs depend on the prevalence of the outbreaks, it was difficult to compare them across different surveillance settings, but they were overall superior to 75%, reaching a maximum at 96.5% for the CuSum algorithm in the study by Brown et al. [30]. Additionally, Carnevale et al. [35] combined multiple sources of alert to estimate the overall number of true positives. This allowed the estimation of sensitivity measures, which varied from 21 to 31% (Fig 3).

Out of the four studies that relied on a panel of experts, three reported inter-rater agreement estimated by Cohen’s kappa. Using binary classifications, Wright et al. [33] and Huang et al. [34] reported good agreement (κ = 0.82 and 0.76 respectively) whereas Carnevale et al [35] reported lower results (from 0.11 to 0.49 on multiple pairs of raters).

Two studies provided estimates of the four traditional performance measures by comparing their algorithms to an “epidemiological” reference standard. Hacek et al. [32] used molecular typing, in a subset of potential alerts selected by the infection control team. Using this method, they reported that traditional surveillance was less specific than simple thresholds and SPC methods, with comparable sensitivity levels (Fig 3).

Alternatively, Tseng et al. [37] evaluated several SPC methods in comparison to the traditional infection control surveillance of vancomycin-resistant enterococcal infections. With the best parameters, the sensitivity and specificity of the algorithms ranged respectively from 77 to 100% and from 83 to 87% (Fig 3).

For these epidemiological approaches, we were able to estimate the inter-algorithm heterogeneity for sensitivity and specificity: according to the I^2^ statistic, this heterogeneity accounted for respectively 83.5% and 67.4% of the overall variability. In the meta-regressions, the study effect explained respectively 100% and 45.33% of this heterogeneity (R^2^ statistics).

#### Derived and simulation approaches

With the ‘derived’ and ‘simulation’ approaches, outbreaks are either statistically defined or identified in advance in the simulated datasets. The detection performances are thus more easily calculated and can even be estimated for different situations by modifying the parameters of the statistical definitions or the simulated datasets. As a result, the studies that used these approaches often reported ranges of performance measures as opposed to point estimates. For example, Kikuchi et al. [40] reported sensitivity and specificity measures for a linear model varying from 80 to 100% and 10 to 95% respectively, depending on the attack rates of the simulated outbreaks, while Skipper [41] reported sensitivity varying from 0 to 100% depending on the type of outbreak simulated, and on the parameters of their multistate Poisson model.

The derived approach also provided a straightforward reference standard for Mellmann et al. [38] to compare different detection methods. They estimated that traditional surveillance was more specific (97.3% vs. 47.3%) but less sensitive (62.1% vs. 100%) than a simple rule based on the frequency of cases. A rule based on both the frequency of cases and the results of molecular typing gave the best overall performance with a sensitivity of 100% and a specificity of 95.2%.

## Discussion

Our literature review yielded 29 studies published between 1981 and 2015 that described algorithms for automated detection of nosocomial outbreaks. Among the different types of algorithms, those that were based on SPC were the most commonly used over the reviewed period. They have been applied for a long time in various fields of quality control and have been recommended for infection control surveillance for about two decades [43]. In the most recent studies, however, scan statistics have been used increasingly, as the popularity of the SaTScan package [44] rose in epidemiological research.

The surveillance scopes and settings in which these algorithms were implemented as well as the methods used to evaluate their performance varied quite noticeably between studies. According to our meta-regressions, the differences between studies explained a large part of the heterogeneity between of the results of this review. This heterogeneity did not allow us to estimate pooled accuracy measures using meta-analysis, and also precluded the comparison of the different algorithm categories. We acknowledge that our literature review might suffer from a selection bias: due to time and material constrains, we only searched one bibliographic database and a single author selected the studies and extracted the data. Despite snowballing, we might therefore have missed relevant studies. However, including more studies would likely further increase the observed heterogeneity.

With so many differences between the studies, it is difficult to draw firm conclusions about the performance of these algorithms for hospital-level surveillance. Nonetheless, they did appear as useful supplements to traditional surveillance carried out by the infection control teams. In fact, as long as one of these algorithms can detect or confirm outbreaks without generating too many false alarms, it can be considered a useful complementary tool. And because infection control professionals can more easily investigate an alert than epidemiologists can in the community, a higher rate of false alerts—and therefore a lower PPV—might be acceptable for hospital-level surveillance. But even if high performances are not required, researchers still need a valid and reproducible framework to evaluate and compare these algorithms.

First, researchers need to determine which performance measures they would like to evaluate, as it will have a great impact on the choice of the evaluation approach. Depending on the study design, estimating the four traditional accuracy measures (sensitivity, specificity, positive and negative predictive value) might be difficult. In the context of outbreak detection, however, this may no be an important issue. Indeed, as “non-outbreaks” are by far more frequent than outbreaks, researchers must deal with what are called imbalanced datasets. In these situations, it has been shown that precision-recall plots, based precisely on VPP and sensitivity, give accurate information about classification performance and are more informative than the traditional ROC curves, based on sensitivity and specificity [45]. We also believe that timeliness is an important feature of any outbreak detection system [46,47], and that it should be evaluated along with the traditional detection performance measures. Although some attempts to evaluate timeliness were made in the studies that we included [30,34,36,41], the results are difficult to interpret and future studies should try to address this question more systematically.

The choice of the evaluation approach is also an important aspect of the framework. The evaluation approaches described by Watkins at al. [9] all offer different insights, but the epidemiological approach is preferable for evaluating detection algorithms in real-life settings, because it provides the best face validity. However, the epidemiological approach also has some drawbacks that should be carefully addressed. First, it might suffer from a lack of statistical power, given the relative scarcity of hospital outbreaks in real settings. Sufficiently large surveillance scopes and periods should thus be available to precisely estimate and compare the algorithms’ accuracy. A second problem with the epidemiological approach is that it does not provide any obvious reference standard, contrary to the simulation and derived approaches. In the present review, two of the nine studies that followed this approach used a reference standard, but neither of them seemed to us fully satisfactory. Tseng et al. used the results of traditional surveillance [37], which, as shown in some of the included studies, is unfortunately an imperfect reference standard. Hacek et al. used molecular typing [32], but did not apply it to every isolate: it was only considered if an alert was generated, and the final decision was taken by infection control professionals. While this strategy is perfectly understandable from an economic point of view, it introduces a partial verification bias, which leads to an overestimation of the detection performances [29].

The main problem with evaluating detection algorithms is that outbreaks do not have a standard definition [47]. According to recommendations for diagnostic studies by Rutjes et al. [48], one appropriate strategy in such situations is to use expert panels. In order to estimate the validity of the panel’s choices, researchers should always report them along with a measure of the inter-rater agreement. Out of the four included studies that used an expert panel, three reported Cohen’s kappa coefficients. But, again, differences in how the experts’ opinions were collected did not allow direct comparison of the results. The two main approaches were: 1) to ask the experts about the probability that a given alert corresponds to a real outbreak, or 2) to ask the experts what actions should be initiated in response to a given alert. Carnevale et al. [35] used a combination of these two approaches, which complicated the validation process and might partly explain why they measured lower inter-rater agreement. The choice between the two approaches should depend on the expected use of the algorithm. For example, if its purpose is to be used as a practical tool for surveillance, the second approach, which focuses on action, is preferable.

Using experts’ knowledge to discern between true and false positives only allows computing VPPs. To estimate the other performance measures, one solution is to use the panel of experts as a real reference standard by asking them to distinguish between “outbreak” and “non-outbreak” periods, as it was done for community outbreak detection in the Bio-ALIRT project [49]. Another solution can be to combine the information brought by various algorithms or data sources. Carnevale et al. [35], for example, gathered the results of different algorithms to estimate the true number of outbreaks and compute sensitivity measures. It is possible however that some outbreaks were missed by all of these sources and that the computed sensitivities were therefore overestimated. In such situations, capture-recapture analyses should be implemented, as proposed in the CDC recommendations for early outbreak detection systems evaluation [47]. Other advanced statistical modeling such as latent class analysis can also combine information from different sources. They are commonly used for diagnostic test evaluation in the absence of a reference standard [48], and it may be interesting to try to use them in the context of outbreak detection.

Another advantage of combining information sources is that it can improve detection performance. For instance, as noted by Carnevale et al. [35], clonal and non-clonal outbreaks have different dynamics and infection control teams may have to use different algorithms to detect each type. This complementarity is well established for control charts: traditional Shewart charts are better for detecting large and sudden deviations from the mean whereas CuSum and exponentially weighted moving averages are better suited for small continuous ones [22].

Several studies also showed that including covariates such as culture site, hospital location and antibiotic resistance can improve the detection performance of the algorithms [23,35,37]. The majority of the studies that we reviewed, however, solely relied on simple time series of cases to trigger alerts. Even though additional sources of data such as electronic medical records and electronic prescriptions might not be readily available for surveillance in all centers, a more thorough investigation of the utility of individual covariates for outbreak detection appears to be another interesting direction for future research.

In addition to a standardized framework, studies on detection algorithms would greatly benefit from a quality assessment tool. We originally wanted to evaluate the quality of the included studies using either the STARD checklist [50] for diagnostic studies or the TRIPOD checklist [51] for prediction model development and validation. Unfortunately, a lot of the items of these tools were not applicable to the context of outbreak detection evaluation. One reason is the variety of the evaluation approaches: the relevant information to be reported is quite different between descriptive, simulation and epidemiological approaches. Another reason is that many items of these checklists address issues about study participants (inclusion, flow, baseline characteristics, adverse events, missing data, etc.), which is not of concern in studies on outbreak detection. Nonetheless, some items of these quality reporting tools address very interesting issues. For example, the TRIPOD checklist differentiates between the development and validation phases for a predictive model. This distinction is important to avoid reporting overly optimistic performances and was only done in seven of the studies that we included. Other examples are the items that relate to statistical power and precision: none of the studies that we included reported a statistical justification of their sample size, and only one of them provided the 95% confidence intervals of their performance measures. Future studies on outbreak detection evaluation should be careful to report these elements.

Undoubtedly, research on the automated detection of hospital outbreak has not yet made the most of the great opportunity offered by modern hospital information systems. More importantly, the evaluation methodology needs to be standardized in order to accurately measure and compare the performances of the detection algorithms. In particular, the different types of algorithm should be compared in a large study using a valid epidemiological reference standard. With these improvements, we believe that these algorithms can become useful decision-making tools for infection control professionals. They can also help to better understand how outbreaks spread within hospitals, ultimately improving patient safety in healthcare.

## Supporting information

S1 Appendix Pubmed search query. (DOCX)

S2 Appendix PRISMA checklist. (DOC)
