# Supplementary material for: Automated detection of hospital outbreaks: A systematic review of methods
Source: PLoS One. 2017 Apr 25;12(4):e0176438. doi: 10.1371/journal.pone.0176438 (PMC5404859; doi:10.1371/journal.pone.0176438)
Supplement: S1 Appendix — (DOCX) [file pone.0176438.s001.docx]

(outbreak OR aberrancy) AND (detection OR detect OR identification OR identify) AND (system OR algorithm OR method OR rule) AND (hospital)
